# Polymer Ring Resonator with a Partially Tapered Waveguide for Biomedical Sensing: Computational Study

**DOI:** 10.3390/s21155017

**Published:** 2021-07-23

**Authors:** Tayebeh Sahraeibelverdi, L. Jay Guo, Hadi Veladi, Mazdak Rad Malekshahi

**Affiliations:** 1 Electrical and Computer Engineering Department, University of Michigan, Ann Arbor, MI 48109, USA; guo@umich.edu; 2 Electrical and Computer Engineering Department, University of Tabriz, Tabriz 5166616471, Iran; veladi@tabrizu.ac.ir; 3 Electrical and Computer Engineering Department, Razi University, Kermanshah 6714967346, Iran; m.malekshahi@razi.ac.ir

**Keywords:** optical sensors, refractive index sensor, polymer waveguide devices, micro-optical devices, sensors

## Abstract

Ring resonators are well-known optical biosensors thanks to their relatively high Q-factor and sensitivity, in addition to their potential to be fabricated in large arrays with a small footprint. Here, we investigated the characteristics of a polymer ring resonator with a partially tapered waveguide for Biomedical Sensing. The goal is to develop a more sensitive biosensor with an improved figure of merit. The concept is more significant field interaction with the sample under test in tapered segments. Waveguide width is hereby gradually reduced to half. Sensitivity improves from 84.6 to 101.74 [nm/RIU] in a relatively small Q-factor reduction from 4.60 × 10^3^ for a strip waveguide to 4.36 × 10^3^ for a π/4 partially tapered one. After the study, the number of tapered parts from zero to fifteen, the obtained figure of merit improves from 497 for a strip ring to 565 for a π/4 tapered ring close to six tapered ones. Considering the fabrication process, the three-tapered one is suggested. The all-polymer material device provides advantages of a low-cost, disposable biosensor with roll-to-roll fabrication compatibility. This design can also be applied on silicon on isolator, or polymer on silicon-based devices, thereby taking advantage of a higher Q-factor and greater sensitivity.

## 1. Introduction

The rapidly increasing demand for precise, fast, compact, and low-cost sensors for healthcare [1], environmental monitoring, and food quality control is attracting substantial interest in optical-based [2] biosensors. Thanks to their relatively high Q-factor and small size, optical microring resonators have long been the subject of research [3,4].

The evanescent field’s interaction with the propagated mode in the surrounding media is the sensing principle of these sensors. Thus, any change in the refractive index alters the effective refractive index and results in a shift in the ring resonance wavelength. Therefore, there is a need for more interaction between the sample under investigation and the evanescent field. However, there is a tradeoff with the Q-factor of the ring. Current waveguide types with increased sensitivity are primarily narrow-width strip waveguides, slot waveguides, and more recently, grating [5] and hybrid waveguides [6]. A strip waveguide with a small cross-section still confines 80% of propagated light; thus, there is not enough interaction with the sample. Meanwhile, slot waveguide-based ring resonators suffer from a low Q-factor because of high loss and because they confine only 30% of the propagated light in the core of the ring [7]. The fabrication and design of grating waveguides are complicated because of the subwavelength feature size and the still relatively low Q-factor of about 7000 [5] for silicon on isolator (SOI) type ring resonators. Although the hybrid waveguides seem to be the most promising design, they have a larger footprint of 2720 μm^2^, which results in a small free spectral range, limited measurable wavelength shift, and consequently a narrower detection range. Moreover, there is a significant loss in the long slot part in a racetrack ring design [7].

**Figure 1:**
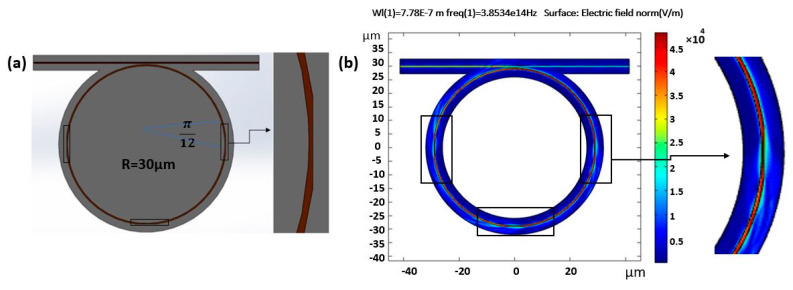
(**a**) Partially tapered ring with a 30 μm radius and (**b**) 3 parts tapered at 0, π, 3π/2; tapered part angle is (π/12).

The present study proposes a partially tapered ring resonator, as shown in Figure 1, to meet the demand for sensitive and high Q-factor devices. The electric field profiles are shown in Figure 1b. Since taper design is quite gradual rather than abrupt and the geometry is round, these cause the spreading of the evanescent field outside the waveguide core even beyond the tapered region. We estimated the confinement in the taper part to describe the effect on sensitivity. Even the sensitivity and figure of merit are higher than our estimation because even after taper parts, we still have more field interaction with surrounding clad. The tapered ring structure has the potential to be manufactured via any ring resonator fabrication methods, such as 193 nm optical lithography [8], electron beam lithography [3], nanoimprint lithography [3], laser interference [9,10], direct laser writing [11], and the two-photon polymerization technique [3] considering the material.

To overcome the limitation of the quality factor of a ring resonator with an asymmetric shape, a sharp asymmetric Fano resonance curve with a larger transmission slope improves the signal-to-noise ratio. Fano resonance is provided by the modified density of states interaction [12] or light-matter bound state to couple the continuum of the electronic density of states to the semiconductor [13] to form Fano resonance. Detail of light-matter interactions and coupling regime is discussed in this reference [14]. Specifically for ring resonators in the past, we have designed and fabricated a polymer microring device by adding two partially reflecting elements in the waveguide. The zero and the unity transmission slope is enhanced compared with a conventional mirroring resonator [15]. In addition, a Composite MZI-Ring [16] and a coupled optical microcavity [17] can make an asymmetric Fano resonance curve. Combining the present study and Fano resonance structure design can potentially allow higher sensitive sensors for future research. Moreover, one can find a time-dependent analysis in this reference [18]. To assume real-time biosensing will require a time scale on the order of a second or tens of seconds, and photon lifetimes in the ring are of the order of nanoseconds. Therefore, the resonator can be considered in a steady state or equilibrium.

## 2. Biosensor Design and Working Principle

A ring resonator comprises a straight and a ring-shaped waveguide in very close proximity so that the propagated field can couple at the ring part. The resonance condition in the ring can be met if the phase of the traveling wave in the ring becomes an integer multiple of 2π, causing constructive interference. For this to happen, the optical path length must also be equal to an integer of a specific wavelength, called the resonance wavelength. Equation (1) shows this relationship, in which M is an integer, λr is the resonance wavelength, $n_{\mathrm{eff}}$ is the effective refractive index of the ring core, and L is the ring’s circumference [19].

(1) $${M\lambda }_{r}=n_{\mathrm{eff}}\times \;L$$

In this regard, the presence of biological elements of interest in the sample under test in the ring’s vicinity causes a change in the cladding’s effective refractive index. This results in a modification of the optical path length and, consequently, a resonance wavelength shift, which can be calculated by [20]:

(2) $$\Delta \lambda_{r}=\frac{\lambda_{r}}{n_{g}}\times \Delta n_{\mathrm{clad}}\times \Gamma_{\mathrm{clad}}≅\frac{\lambda_{r}}{n_{\mathrm{eff}}}\times \Delta n_{\mathrm{clad}}\times \Gamma_{\mathrm{clad}}$$

where $\lambda_{r}$, $\Gamma_{\mathrm{clad}}$ and $n_{g}$ are the resonance wavelength, field confinement factor in the clad, and group index, respectively. Assuming a slight wavelength shift, which results in neglected dispersion, we can rephrase Equation (2) with $n_{\mathrm{eff}}$ [2]. Also, $\Delta n_{\mathrm{clad}}$ is the clad refractive index, which is directly proportional to the refractive index change in the sample under test. $\Gamma_{\mathrm{clad}}$, which is the field leakage in the clad is a crucial parameter in ring resonator biosensor design. The field leakage in the cladding region is defined by the ratio of the surface integration of the electric field penetrating the cladding domain to the total electric field in the entire domain. Sensor sensitivity is defined by [21]:

(3) $$S=\frac{\Delta \lambda_{r}}{\Delta n_{\mathrm{eff}}}=\frac{\lambda_{r}}{n_{\mathrm{eff}}}\times \frac{\Delta n_{\mathrm{clad}}}{\Delta n_{\mathrm{eff}}}\times \Gamma_{\mathrm{clad}}$$

Therefore, increasing the leakage in some parts of the ring resonator makes it possible to have a more significant wavelength shift and a more sensitive device. In addition, maintaining a high Q-factor leads to an improved limit of detection (LOD), which can be defined by [21]:

(4) $$\mathrm{LOD}=\frac{\mathrm{FWHM}}{S}$$

The full width at half the maximum of the device’s transmittance curve in the resonance condition is called FWHM. The Q-factor defines the amount of energy stored in the resonator to the energy lost in the ring resonator per cycle. Q-factor also describes the ratio of resonance wavelength to FWHM, as illustrated in Equation (5).

(5) $$Q_{\mathrm{actor}}=\frac{\lambda_{r}}{\mathrm{FWHM}}$$

The typical measurement setup resolves the resonance wavelength shift by detecting the intensity change on a set measurement wavelength on the transmittance curve’s steepest angle. Hence, it is desirable to have a high Q-factor and simultaneously a sensitive device for biosensor applications. Our proposed design makes this more feasible.

## 3. Materials and Methods

Polymers are used for all the core, clad, and substrate materials, adding the advantage of having low-cost and disposable sensors thanks to the potential for roll-to-roll fabrication [21]. All-polymer ring resonator structure brings less temperature-sensitive devices due to close thermal expansion and thermo-optic constants compared to polymer on silicon substrates. To develop the idea, we compared a strip waveguide-type ring with the proposed partially tapered design with a similar cross-section and ring radius made of the same material. The simulation was performed using the 2D finite element method based on the wave optics module of COMSOL Multiphysics and a frequency domain in the 780 nm central wavelength. The refractive index assigned for the Epo-Core negative tone photoresist was equal to 1.57 for the core and polydimethylsiloxane (PDMS), with 1.417 for the substrate and cladding [22].

**Figure 2:**
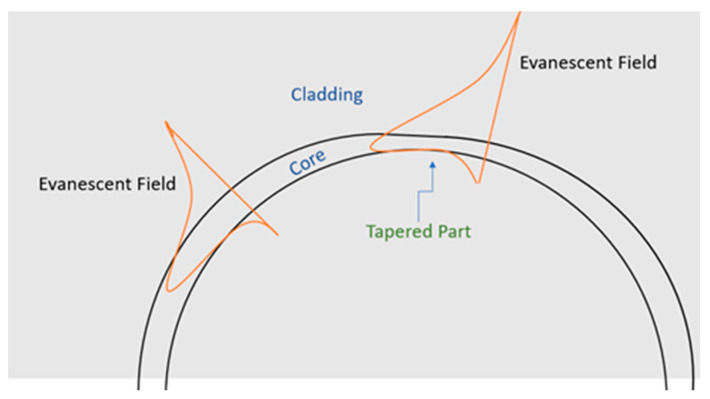
Illustration of evanescent field propagation in the tapered part compared to the strip part.

The proposed idea with three tapered parts is shown in Figure 1. Each part is equal to π/12 at 0, π, and 3π/2. This configuration provides an equal chance for the agent of interest to be detected in the sample under test. The waveguide cross-section was 550 nm in width and 1 µm in height. The tapered part was half the width and was equal to 275 nm with the same height, resulting in less electric field confinement in the core and more leakage in the surrounding clad. The tapered parts in the ring exhibited less field confinement and a longer evanescent field in the cladding part, as shown in Figure 2.

**Figure 3:**
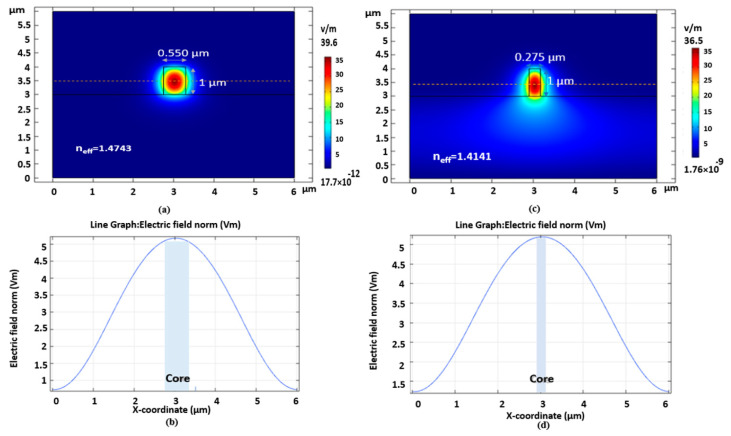
Mode profile, normalized E−field distribution of the TE fundamental mode, and the calculated effective index in the strip waveguide parts (**a**,**b**) and tapered parts (**c**,**d**).

The idea provides a more remarkable resonance wavelength shift with the same refractive index change due to the existing biological element of interest in the sample under test in the cladding region. Figure 3 illustrates the mode profile, normalized E-field distribution of the TE fundamental mode and calculated effective index in the proposed device’s tapered parts and strip waveguide parts.

Each tapered part’s angle section was equal to 15°, or π/12. The effective index of the new ring geometry was calculated by:

(6) $$n_{\mathrm{eff}}\times L_{\mathrm{ring}}=n_{\mathrm{eff}\;\mathrm{strip}}\left(2\pi R-L_{\mathrm{taper}}\right)+L_{\mathrm{taper}}\times n_{\mathrm{eff}\;\mathrm{taper}}$$

where $L_{\mathrm{ring}}$ is the total ring circumference, $L_{\mathrm{taper}}$ is the tapered arc length, $n_{\mathrm{eff}\;\mathrm{strip}}$ is the effective index of the strip waveguide parts, and $n_{\mathrm{eff}\;\mathrm{taper}}$ is the effective refractive index of the tapered parts. Each tapered part size was equal, and we had three tapered parts; thus, the total length of the tapered parts was equal, and the calculated effective index was 1.474. The effective refractive index concerning the ring waveguide type was 1.4743, 1.4742 and 1.4741 for strip waveguide; π/12 one part tapered, and two-part tapered, respectively. To calculate the resonance wavelength shift in Equation (2), we needed to have the value, which is the confinement factor of the cladding field based on Equation (2). Regarding our partially tapered ring design, we calculated the whole ring leakage factor by:

(7) $$\Gamma_{\mathrm{Clad}\;\mathrm{ring}}=\frac{n}{24}\times \Gamma_{\mathrm{taper}}+\left(1-\frac{n}{24}\right)\times \Gamma_{\mathrm{strip}}$$

where *n* is the quantity of the tapered part in our design, equal to π/12 for one tapered part and, $\Gamma_{\mathrm{taper}}$, $\Gamma_{\mathrm{strip}}$ are the factors of tapered parts and strip parts, respectively.

The ring clad leakage factor ($\Gamma_{\mathrm{Clad}\;\mathrm{ring}}$) is the fraction of the light field spreading to the cladding region, which is defined by the ratio of the surface integration of the electric field penetrating the cladding domain to the total electric field in both core and cladding domains.

## 4. Discussion

**Figure 4:**
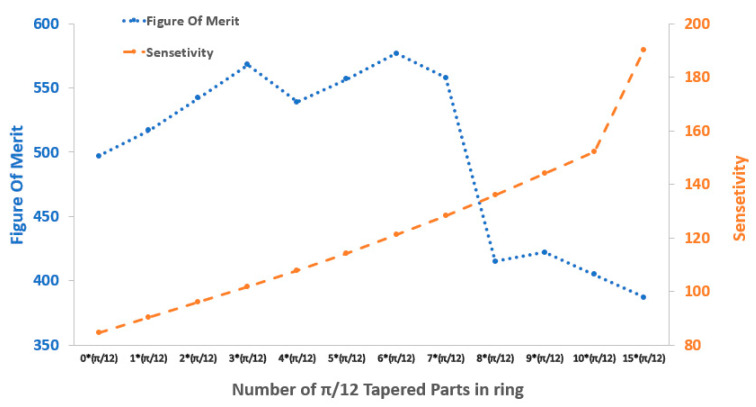
Sensor sensitivity and Figure of Merit (FOM) versus ring design type (* is cross).

Our model presents $\Gamma_{\mathrm{strip}}\mathrm{equal}$ to 0.16 for the strip waveguide parts and a $\Gamma_{\mathrm{taper}}$ equivalent to 0.43 for the tapered parts, calculated via mode profile simulation in COMSOL. Have $\Gamma_{\mathrm{Clad}\;\mathrm{ring}}$ versus ring type, we can calculate ring sensitivity. The effective refractive index and cladding confinement factor were calculated from previous parts. Sensor sensitivity and Figure of Merit (FOM) versus ring design type is presented in Figure 4.

We swept the number of parts from zero to 15 (π/12) parts. The result for sensitivity showed an increase of sensitivity in the expense of reduction of the Q-factor to find the optimum point; we calculated Figure of Merit (FOM), showing an improvement from one taper to seven tapers compared to the case of no taper. With more detail, we saw that we had the highest FOM for three- and six-taper designs, which also were close in FOM value. Therefore, we suggest three tapers as the best FOM by considering the fabrication process since after seven taper parts, the trend of losing the quality factor was evident and sharp. We can anticipate the continuous reduction of quality factor and FOM after seven tapered parts due to excess light leakage.

**Figure 5:**
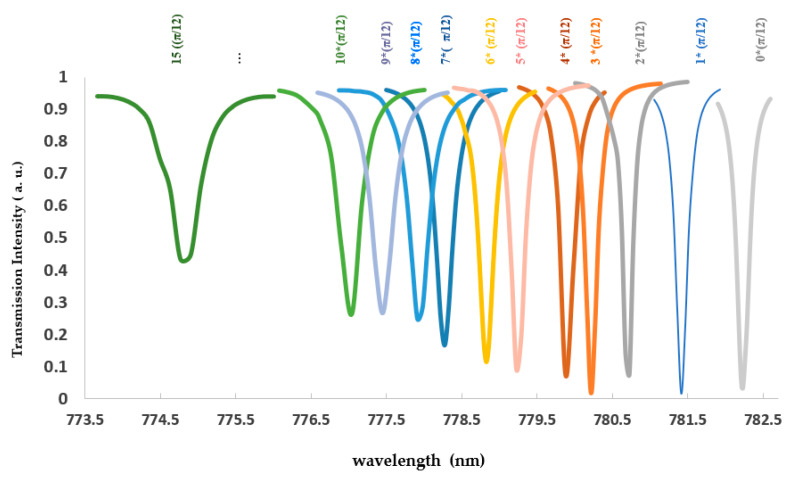
Transmittance curve for ring designed with zero tapered parts to 15 * (π/12); (* is cross).

Figure 4 demonstrates that the partially tapered ring design obtains a sensitivity improvement from 84.5 nanometers per refractive index unit (RIU) for the strip zero taper waveguide to 101.74 [nm/RIU] for the 3*π/12 (π/4) tapered ring, revealing an enhancement of more than twenty percent. The transmittance curve for the ring designed with zero tapered part to parts is presented in Figure 5. The Q-factor is calculated from the ratio of the resonance wavelength to the FWHM and is shown in Table 1.

Thus, our all-polymer ring resonator with a resonance wavelength of 782,220 nm, considering 2 nm free spectral range for demonstration in Figure 5 with an FWHM of 0.170 nm, resulted in a quality factor of 4.60 × 10^3^, using Equation (5), whereby the FWHM was extracted from the 1D plot of the transmittance curve from Figure 5, which is provided by this command (abs (comp1.ewfd2. S21^2^)) from the simulated model. In the same way, FWHM of 0.179 nm and resonance wavelength of 780,180 nm was obtained for the π/4 tapered ring. The quality factor of 4.36 × 10^3^, indicating about a six percent reduction compared to the strip waveguide ring, is represented in Figure 5. In addition, based on Equation (4), LOD is the ratio of FWHM to sensitivity. The partly tapered design in this work resulted in an improved LOD compared to a similar strip design, indicating this design’s potential in enhancing LOD.

Furthermore, to obtain the resonance wavelength from the 2D wave optics frequency domain of the COMSOL model, a swept wavelength of 777–782 nm was used. Therefore, using Equation (3), we could calculate the device sensitivity for each designed type presented in Figure 4. The figure of merit (FOM) can be defined by the relation between the sensor sensitivity and the related FWHM, as illustrated in Equation (8) [6].

(8) $$\mathrm{FOM}=\frac{S}{\mathrm{FWHM}}$$

This paper aims to introduce a novel ring resonator design that brings higher sensitivity with relatively small Q-factor reduction, which results in FOM improvement, increasing field fraction toward cladding regions where biomarkers of interest exist. We studied this goal, adding more tapered parts starting from one tapered part of π/12 size to fifteen π/12 parts. We began by pi/12 to have a sensible change in the sensitivity from 84 to 90 from no taper to one taper to study the effect of adding tapers on the ring sensitivity while observing quality factor reduction.

We considered that more tapered parts would cause too much light leakage and affect the Q-factor, and then there would be no further gain in detection. Since there is a definite tradeoff between the sensitivity and fabrication complexity, and the attainable figure of merit for introducing the taper, we did not design with a smaller taper length and more quantity. 

The partially tapered design can also be applied to SOI-based devices with a telecom wavelength and lower loss, offering a potentially smaller footprint in addition to a higher Q-factor. Furthermore, higher Q-factors can be obtained by rings with larger radii because of less bending loss. However, this results in a larger footprint. Moreover, SOI or polymer on silicon-based devices can obtain higher Q-factors thanks to the relatively more considerable refractive index difference between the core and cladding materials. A resonance wavelength of 780.180 nm was obtained for the three-tapered parts design. Therefore, by implementing Equation (8), we calculated the FOM of 565 for three-tapered-parts ring waveguides versus 497 calculated for strip waveguide one.

## 5. Conclusions

We propose a relatively simple design with a small device footprint of about 100 μm with a waveguide cross-section of 550 nm in width and 1 µm in height. A key advantage of this design is its improved sensitivity and enhanced LOD, with an opportunity for less Q-factor reduction as a known tradeoff with sensitivity. The introduced design principle is based on more significant field interaction with the sample under test by partially tapering the ring waveguide. A relatively small Q-factor reduction in a π/4 partially tapered ring from 4.60 × 10^3^ to 4.36 × 10^3^ improved the sensitivity from 84.6 to 101.74 [nm/RIU]. The FOM obtained for the partially tapered ring improved from 497 to 565 compared to a strip ring of the same size. By parametric study, after adding seven-taper parts, the quality factor reduced fast. It can be predicted that more tapered parts cause more loss and affect FOM as well. The optimum found by the calculated Figure of Merit started improvement from one taper to seven tapers versus no taper one. The highest FOM was achieved for three- and six-taper designs, and they were very close in amount. Here we suggest a three-taper design as the best FOM by considering the fabrication process.

A ring radius of 30 μm provides a free spectral range (FSR) of 2 nm, a good range for biosensing applications. The ring and waveguide used in this work are all-polymer, leading to roll-to-roll fabrication feasibility and the potential for low-cost disposable biosensors. The proposed idea here can also be applied to SOI-based ring resonators. Due to the silicon waveguide’s high refractive index contrast using silicon dioxide (SiO_2_), less bending loss and higher Q-factors can be obtained. Furthermore, using the telecom wavelength, which is 1500 nm for SOI-based devices versus 780 nm as the designed wavelength in polymer-based ones, leads to a higher Q-factor, derived from Equation (5). 

The experimental study is the future work plan, in which other students will test the predictions experimentally. We will complete the fabrication of this design idea as a future step toward achieving this project.

## Figures and Tables

**Table 1:** Quality factor versus number of π/12 tapered parts in the ring.

Number of π/12 Taper	0	1	2	3	4	5	6	7	8	9	10	15
Q-Factor	4600	4505	4440	4360	3900	3801	3709	3384	2363	2338	2078	1592

## Data Availability

No new data were created or analyzed in this study. Data sharing is not applicable to this article.
